# Racial Minority Doulas' Perceptions of Hospital Team-Based Care

**DOI:** 10.1097/NMC.0000000000001125

**Published:** 2025-08-13

**Authors:** Leanne T. Burke, Elaha Noori, Catherine Pham, Vina Heng, Candice Taylor Lucas, Yuqing Guo

**Affiliations:** **Leanne T. Burke** is an Associate Clinical Professor in Sue & Bill Gross School of Nursing at the University of California, Irvine, CA. The author can be reached at ltburke@hs.uci.edu; **Elaha Noori** is a Medical Student in the School of Medicine at the University of California, Irvine, CA. The author can be reached at noorie@hs.uci.edu; **Catherine Pham** is a Medical Student in the School of Medicine at the University of California, Irvine, CA. The author can be reached at cathetp3@hs.uci.edu; **Vina Heng** is a Nursing Science Student in Sue & Bill Gross School of Nursing at the University of California, Irvine, CA. The author can be reached at vsheng@uci.edu; **Candice Taylor Lucas** is an Associate Clinical Professor in the School of Medicine, Department of Pediatrics at the University of California, Irvine, CA. Dr. Lucas can be reached at taylorce@hs.uci.edu; **Yuqing Guo** is an Associate Professor in Sue & Bill Gross School of Nursing at the University of California, Irvine, CA. Dr. Guo can be reached at gyuqing@hs.uci.edu

**Keywords:** Doulas, Ethnic and racial minorities, Health inequities, Labor and delivery, Maternal mortality, Patient care team, Pregnant women

## Abstract

**Introduction:**

Doulas are skilled paraprofessionals who provide supportive care to pregnant women and birthing people resulting in improved outcomes. However, conflicts persist between health care providers and doulas in hospital-based maternity care teams. Few studies have addressed this phenomenon from the doulas' perspective, particularly doulas from racial and ethnic minority backgrounds.

**Methods:**

This qualitative study used individual semi-structured interviews to explore the experiences and perspectives of perinatal doulas caring for pregnant women from various backgrounds. Transcriptions were analyzed.

**Results:**

Seven doulas participated, five of whom self-identified as Black or African American. Three themes were identified: *Barriers to Including Doulas in Team-Based Care*, *Facilitators to Improving Interdisciplinary Collaboration*, and *Educational Needs and Support*. Doulas emphasized the need for mentorship and support for novices transitioning to hospital settings, particularly when caring for high-risk patients.

**Conclusions:**

Doulas are integral in advocating for pregnant women from diverse backgrounds and helping them to navigate complex health care systems. Given the inequities affecting Black and African American maternity patients, incorporating racial minority doulas in team-based care is critical. Career development and mentorship can help novice doulas integrate smoothly into hospital environments.

## Abstract 2

In this study doulas from racially diverse backgrounds described their experiences in working in the hospital maternity setting with other health care professionals who care for women during pregnancy, labor, and postpartum. Facilitators and barriers to effective care were discussed as well as opportunities for enhanced education and better integration into the maternity health care team.

**Figure:**
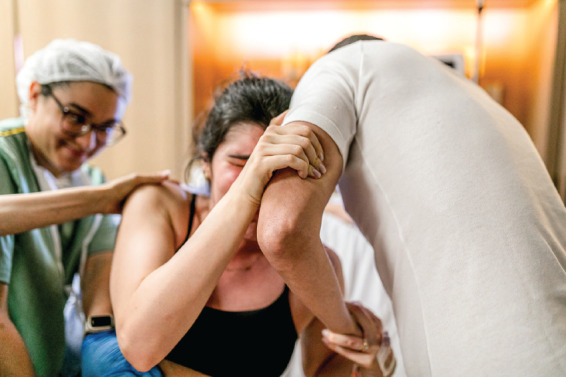


In the United States, maternal mortality rates remain high compared with other high-income countries such as New Zealand, Germany, and the United Kingdom, despite advancements in modern medicine and higher spending on health care per person (Gunja et al., 2024). The past 2 decades have also shown increasing trends of maternal mortality rates among all racial and ethnic groups (Fleszar et al., 2023). Doula care is one promising avenue for lessening these maternal health disparities, yet it is far underutilized in the modern U.S. health care system (Van Eijk, Guenther, Jopson, et al., 2022), despite being supported by both nursing and medical professional organizations (American College of Obstetricians and Gynecologists [ACOG], 2019; Association of Women's Health, Obstetric and Neonatal Nurses [AWHONN], 2018).

Doulas are paraprofessionals who provide continuous, intimate care to their clients before, during, and shortly after childbirth (National Doula Certification Board [NDCB], n.d.). Their primary role is to offer emotional and social support to pregnant women throughout their childbearing year, including pregnancy, labor, birth, and the transition into postpartum (NDCB, n.d.).

Evidence has found that those with continuous labor support had positive perinatal outcomes, such as shorter lengths of labor, increased chance of spontaneous vaginal birth, decreased use of epidural analgesia, improved 5-minute Apgar scores, and increased patient satisfaction (Bohren et al., 2017; Saigh et al., 2024). The positive effects of continuous birth support, particularly from doulas, have been shown to be far greater among women who were unmarried, primiparous, low-income, or from marginalized minority populations (Marudo et al., 2023; Safon et al., 2024; Thomas et al., 2017; Vonderheid et al., 2011). In an observational study of low-income birthing mothers, doula-assisted mothers were four times less likely to have a low-birth-weight baby, two times less likely to experience adverse birth complications, and significantly more likely to initiate breastfeeding (Gruber et al., 2013; Sobczak et al., 2023). The lower rate of birth complications also serves as a potential source of reduced health care costs.

According to the *Listening to Mothers III survey*, approximately 6% of U.S. women received supportive care from a doula during labor (Declercq et al., 2014). Although Medicaid coverage is increasing for doula care, there continue to be barriers such as low reimbursement rates and a cumbersome process to get access and coverage (Kozhimannil & Hardeman, 2015; Marshall et al., 2024). Although Medicaid programs are disproportionately used by racial and ethnic minority populations, there are few studies understanding doulas' perspectives, particularly those from racial and ethnic minority backgrounds (Declercq & Zephyrin, 2020; Mallick et al., 2022; Markus et al., 2013). The purpose of this study was to explore the experiences and perspectives of racial minority perinatal doulas caring for women from a variety of socioeconomic, racial, and ethnic backgrounds throughout pregnancy, labor, birth, and postpartum.

## Methods

We used a qualitative study design with a semi-structured interview guide and a demographic survey, both developed by the research team based on a literature review (Attanasio et al., 2021). After Institutional Review Board registration, recruitment began through email and in-person flyer distribution. The Academic Health Center's Diversity, Equity, and Inclusion Initiative members shared the flyer with groups such as the Childbirth and Postpartum Professional Association (CAPPA), an international certification organization for doulas and educators. The research team attended the Multicultural Health Expo to reach regional doulas. This purposeful recruitment approach ensured diverse perspectives, particularly from doulas serving high-risk racially and ethnically diverse communities with disproportionately high morbidity. Eligibility criteria included adults over 18 with experience as a perinatal doula for at least three pregnant women in hospitals, birth centers, or home settings.

Eight eligible doulas were scheduled for interviews within 3 months using community support. Challenges included planning interviews around unpredictable work schedules and ensuring completion of demographic surveys. Participants provided verbal consent and completed a demographic survey. The research team conducted virtual interviews using a semi-structured guide. Example questions included: “Can you describe your experience working with other birth team members, such as nurses or other health care providers, to provide effective support to your clients?”; “What additional training and support do you think would have helped you be prepared for your role as a doula?”; and “What are some of the essential skills that you think every doula needs to be effective if they work primarily in a hospital setting?” Participants received a gift card of $40 to show appreciation of their time.

Recorded interviews were then transcribed and cross-referenced with field notes. When eight participants had been interviewed, researchers noted that no new insights emerged, and no further themes were identified on analysis of the interviews, suggesting data saturation had been met. At that time, the researchers deemed continuing to seek further participants would not yield new information, therefore, recruitment ended (Elo & Kyngäs, 2008; Sebele, 2024). Transcriptions were imported into ATLAS.ti's online platform for inductive content analysis that focused on using the human experience and qualitative data itself to provide new insights and a practical guide to action (Elo & Kyngäs, 2008). Four steps were involved in analyzing the interviews: 1) each transcription was reviewed entirely to understand a participant's experiences; 2) meaning units were identified to develop the codes as well as the codebook; 3) similar codes were clustered as categories; and 4) categories were sorted and abstracted into themes. The research team discussed and reached agreement on codes, categories, and themes (Elo & Kyngäs, 2008).

## Results

### Demographic Characteristics

Eight perinatal doulas who met inclusion criteria were interviewed over 3 months, beginning in August of 2023. Of the eight interviews, seven were analyzed. One interview was excluded due to audio issues. Of seven doulas, five self-identified as Black or African American, one as Asian, and one as “other”. The average age of the doulas was 41.8, ranging from 23 to 64 years. Two reported attending some college or technical school, three had a bachelor's degree, and two obtained a master's degree or above. Six worked as a doula for over 3 years, six were community-based doulas, and one was hospital-based. All doulas had experience working with several types of health care providers (such as registered nurses, midwives, and obstetricians) and across various settings. For example, all had worked in hospitals (six in teaching, five in community), four had worked in both birth centers and home birth. Although all but one doula currently worked in California, six also had previous doula experience in other states, including Arizona, Texas, Illinois, Wisconsin, and Missouri.

### Themes

Thirty-six individual codes were identified and organized into 14 categories and three themes, including: *Factors that Improve Interdisciplinary Collaboration, Barriers to including Doulas in Team-Based Care*, and *Educational Needs and Support*.

#### Factors that Improve Interdisciplinary Collaboration

**Understanding of Health Care Environment.** Five doulas expressed having knowledge of a hospital's policies and procedures and knowledge of medical terminology would be helpful to communicate with patients and health care providers. They emphasized the importance of hospital orientation to enhance their effectiveness and comfort in clinical settings. This familiarization would improve understanding of the health care environment and promote better collaboration with the health care team. One doula highlighted the significance of this orientation, *knowing what to do in the crisis situation if there's ever something like code blue, rapid response, knowing what to do and how to behave in situations like that.* Doulas seek to gain specific knowledge through orientation, demonstrating their commitment to professional integration within the health care team.

**Mutual Respect Between the Doula and Birthing Team.** Six doulas emphasized the importance of maintaining respectful relationships with the health care team to enhance their clients' experiences. They reported that a shared understanding among the birth team fostered mutual respect and a collective commitment to achieving the birth plan goals. This alignment of purpose between doulas and health care providers created a more cohesive and supportive environment for the birthing process. One participant noted, *I don't get in the way of what they're trying to do professionally and safely. … my role is to be there for … that mom, encouragement for that mom … I'm not trying to be the doctor or nurse.*

Doulas reported that clear, positive interactions led to several benefits, including enhanced teamwork, reduced role confusion, and fewer misunderstandings between doulas and health care providers. *Effective communication is giving and receiving input and finding the best outcome for the patient. We … [must] listen and digest what we hear. … if we do that … the outcome will be better—there won't be any miscommunication*. Doulas also emphasized that establishing clear boundaries of their scope of practice and defining their role is crucial for successful collaboration. They noted that when their non-medical role is well-defined, it prevents role confusion and improper reliance on doulas for medical advice. Through clear communication, doulas can effectively advocate for patients' wishes and educate team members, fostering a more cohesive care environment. For example, one doula explained how she introduced herself to the health care providers and her role in the birthing space in this way, *just saying that I'm not here to take… any jobs or [break] any rules. But I'm here to support the patient along with supporting you*.

#### Barriers to Including Doulas in Team-Based Care

**Lack of Understanding of Doula Role by Health Care Team.** All doulas reported challenges stemming from health care teams' lack of understanding about their role, which hindered their ability to support patients effectively. Doulas also perceived resistance to their presence, leading to the dismissal of their value by some health care team members. These barriers highlight the need for improved education and integration of doulas within the health care system to enhance collaborative care during childbirth. *I am trying to ensure that my client gets the information, that you're being truthful and transparent with my client and my client takes that information and makes an informed decision that's going to work best for their family*.

**Lack of Communication.** Six doulas reported that poor or inconsistent communication with health care team members can negatively affect the birthing experience, especially when opinions differ on labor care and support. For example*, The hospitalist walks in, and she was on all fours… So, the doctor walks in and goes, “I can't deliver a baby like this.” And … I turned and said, “well get somebody in here who can.”* This lack of communication and openness to other approaches was described as leading to a more challenging birthing environment.

**Lack of Rapport/Trust.** Three doulas reported significant challenges due to a lack of rapport and trust from health care team members. This barrier stems from strained relationships, often resulting from misunderstandings or different perspectives on doulas' roles in the birthing process. Some doulas expressed concern that hospital staff may view them as outsiders, leading to skepticism or resistance toward their presence. *When a mother's desires for her birth are being expressed, sometimes you get a lot of pushback being the doula. It's like ‘you don't know what you're talking about, we know what's best, and this is what's going to happen*. Consequently, doulas reported feeling marginalized or undervalued by health care professionals who prioritize medical expertise over emotional support during childbirth.

**Disconnect between Doulas and the Health Care Team.** Five doulas reported personal challenges in their interactions with health care professionals during the birthing process, whereas the other two shared experiences of fellow doulas they know. The primary issues identified were a lack of awareness among health care workers about doulas' roles, expertise, and established relationships with their clients. This knowledge gap often resulted in doulas being overlooked or excluded as essential members of the birth team, creating a disconnect in collaborative care between doulas and the health care team. *Some medical staff will feel like…you're there for the wrong reasons or you're there to kind of be oppositional to them, kind of you know, making sure like where that professionalism comes in respecting their position and what they're doing and that way usually they'll be more respectful of your position.*

#### Doula's Educational Needs and Support

**Advancing Competence.** All the doulas identified key educational needs, encompassing both foundational knowledge and broader skills, that would enhance their education and professional development. Specific topics of focus included: understanding of pregnancy, labor, and postpartum processes, labor support techniques, lactation, infant care, communication, emotional self-regulation, patient advocacy, cultural competence, and compassion. Three of these topics were highlighted by one participant. *I would say a lot of doulas need to work on their communication skills, management, and emotional self-regulation*. The doula experience indicates that these skills, encompassing both content-specific skills and transferable skills such as communication, teamwork, and problem-solving, are critical for success across various settings, extending beyond clinical skills.

**Career Enhancement and Mentorship.** Career enhancement and mentorship are crucial components of an effective doula education program. Two doulas stressed the need for business education, including billing and reimbursement skills, *It's not just about doing people favors because you like them*. Three others also included ongoing education and mentorship opportunities for novice practitioners.

Some doulas face financial challenges. *My time is valuable… maybe I charge this fee, but am I taking into account how much it will cost for me to have childcare? Or am I telling people “I have no issue with coming to you wherever you are,” but I'm driving 2 hours away. That's gas and wear and tear on my car*. The emphasis on continuing education and mentorship in doula education programs by six of the doulas reflects the recognized benefits of ongoing support for novice practitioners. This approach helps new doulas navigate challenges and apply theoretical knowledge in practical settings. *There should be mentorship for at least a year. Because … as a new doula, you're going by textbook. … textbook does not always translate into real life.* One doula suggested adopting a preceptorship model or support group framework to address the developmental needs of novice doulas transitioning to independent practice.

## Discussion

This study contributes to existing literature by identifying factors affecting the integration of racial minority perinatal doulas into hospital care settings. Our findings highlight the importance of a comprehensive understanding of the health care environment and professional roles when incorporating doulas into team-based perinatal care. These findings align with and expand upon previous research. Shubert et al. (2024) found success in integrating culturally congruent doulas into an urban hospital maternity setting. New mothers with doulas were more likely to attend postpartum visits than those who did not have a doula (Shubert et al., 2024). Young (2022) identified challenges most White doulas face in accessing hospital resources and providing competent care. Richards and Lanning (2019) found that operating room orientation and role overview significantly aided volunteer doulas in cesarean birth settings, improving their integration into collaborative care. Our study extends this evidence, suggesting that familiarity with the hospital environment and clinical roles may also enhance racial and ethnic minority doulas' comfort and competence in interprofessional team settings.

Our findings emphasize that effective communication is crucial for developing trust and clear professional boundaries within the perinatal care teams. Our results corroborate a previous qualitative study with primarily White doulas sharing that communication is an essential factor in promoting interprofessional collaboration (Louis-Jacques et al., 2024). Although communication difficulties and role confusion were identified as familiar challenges, this study extends the literature by suggesting that improved communication and shared understanding of well-defined interprofessional roles are important team-building strategies for racial minority doulas. These strategies may foster mutual respect and collaborative care between these racial minority doulas and health care providers.

Racial minority doulas highly value educational opportunities and continued professional growth. Their insights pointed out educational gaps that need to be bridged to adequately serve patients, as well as emphasized professional development as one crucial strategy to integrate doulas into perinatal care teams. Our results are consistent with evidence that clinical knowledge and skills, such as understanding labor dynamics, common medical interventions, and lactation knowledge, are fundamental to equip doulas (Lanning & Klaman, 2019). Our study supports recent research that it is critical to provide emotional support and mentorship to reduce burnout and increase retention for both novice and experienced doulas of racial and ethnic minorities (Darvish et al., 2024; Louis-Jacques et al., 2024). This approach aligns with established mentorship models in academic and professional settings, which have been shown to support career development and skill acquisition (Doula Apprentice Program, n.d.; Doula Circle, n.d.).

### Limitations

This study's participants, representing various educational levels, doula practice backgrounds, and regions across the United States, enhance its transferability. However, the findings may be most applicable to Black or African American doulas with over 3 years of experience in similar regions, primarily in California and teaching or community hospital settings.

## Clinical Implications

Despite growing doula programs, more evidence-based racial and ethnic minority doula education programs are needed as one of the critical strategies to address maternal disparities across the nation (Van Eijk, Guenther, Kett, et al., 2022). Our study can be used to inform development of a program that will facilitate racial minority doulas to be integrated into hospital-based team care. This type of program can provide continued support to address racial minority doulas' needs beyond basic competence, which is crucial for long-term sustainability. Adding racial minority doulas to the interprofessional team can promote healthier outcomes of patients from diverse backgrounds through improving health literacy and improved communication (Shubert et al., 2024; Thurston et al., 2019). Therefore, integrated doula care is one approach to addressing maternal health disparities.

### Acknowledgments

The study was supported by the University of California, Irvine Undergraduate Research Opportunities Program and the US DHHS Award #1 CPIMP231376-01-00: Black PEARL (Promoting Equity Anti-Racism and Love) Model: Systemic Integration of Community Maternal Support Services to Achieve Birthing Justice, Equity, and Joy for BIPOC Families.

The authors are thankful for Dr. Betty Ferrell who reviewed and edited the manuscript.

## Clinical Implications

- It is important to recognize the pivotal role racial minority doulas play in maternal health equity.
- Racial minority doulas' support is vital for high-risk pregnant patients, as they often require extra support to navigate complex health care systems and address systemic disparities.
- It is essential to eradicate the barriers to including racial minority doulas in team-based perinatal care and delineate the strategies they can use to navigate these challenges.
- By fostering better understanding, communication, and trust between doulas and health care providers, we can ensure that pregnant women from diverse racial and ethnic backgrounds feel supported and advocated for throughout the childbirth continuum.
- Interprofessional health care provider and doula team-based care can lead to a more inclusive and patient-centered approach to perinatal care that prioritizes the needs and preferences of all pregnant women.
- Increased Medicaid coverage and support is needed for doulas to become more widely used.
